# Beyond sex: the effects of testosterone on visuomotor performance in men and women

**DOI:** 10.3389/fnhum.2025.1718846

**Published:** 2026-01-12

**Authors:** Nicole Smeha, Diana J. Gorbet, Heather Edgell, Alison K. Macpherson, Lauren E. Sergio

**Affiliations:** School of Kinesiology and Health Science, York University, Toronto, ON, Canada

**Keywords:** cognition, cortex, hormones and performance, MRI, visuomotor ability, working-age adults

## Abstract

**Introduction:**

The ability to perform visually-guided motor tasks requires the transformation of visual information into programmed motor outputs. When the guiding visual information does not align spatially with the motor output, the brain processes rules to integrate somatosensory information into an appropriate motor response. Performance on such rule-based, “cognitive-motor integration” (CMI) tasks has been shown to be affected by sex, age, and in several neurologic conditions. The present study sought to (1) expand on these findings by examining whether such performance differences are related to levels of sex steroid hormones, and (2) characterize the relationship between hormone levels and any structural differences in brain regions responsible for complex motor control.

**Methods:**

Thirty-six healthy individuals (18 females) underwent MRI scanning to acquire anatomical brain images. They performed two touchscreen-based eye–hand coordination tasks, including a standard direct interaction task and one which involved CMI; target location and motor action were dissociated in the CMI task. Saliva samples collected on the day of testing were used to determine estrogen, progesterone, and testosterone levels.

**Results:**

Multiple regression analyses revealed age to be a small but significant predictors of performance in a CMI condition with visual feedback reversal. We found that after accounting for this age effect, testosterone was a significant predictor of CMI performance in this group. We also observed that the relationship between testosterone levels and complex performance was related to grey matter thickness and volume in visuomotor control regions.

**Conclusion:**

These data suggest that underlying brain networks controlling simultaneous thought and action may differ as a function of sex steroid hormone concentrations, and that small performance declines emerge in the working-age years.

## 1 Introduction

Goal-directed movement is a central aspect of human living. Most of our daily activities require interactions with objects in our environment and consequently rely on the brain’s ability to transform visual and proprioceptive information into coordinated action. These situations, in which we reach towards a viewed object, are known as ‘standard’ or coupled reaches, and represent our brain’s default mode of reaching. Such direct eye-hand coordination is common across species and is crucial for basic survival. However, many of our daily activities increasingly require integration of thought and action, such as applying context-specific rules to guide movement. For instance, the use of a computer mouse incorporates a simple yet concrete rule: move the mouse forward in the horizontal plane to orient the cursor vertically. Correctly performing such ‘non-standard’ movements draws on cognitive functions such as attention and rule-based processing, as well as sensory contributions that include vision and proprioception (Sergio et al., 2009; Wise et al., 1996). Thus, functional communication between the brain regions involved with cognition, sensation, and movement control has a central impact on people’s quality of life and independence. Over the course of a lifetime, the brain undergoes continuous and dynamic changes that can disrupt these systems at multiple levels. For example, aging is associated with increases in processing speed (Finkel et al., 2007), as well as impairments in attention and perceptual abilities (Bendayan et al., 2017; De Fockert et al., 2009). At the same time, older adults begin to move more slowly and less accurately than they once did (Rabbitt, 1997; Cabeza et al., 2002). During standard visually-guided movements, older adults are less accurate and perform more reaching errors, exhibit altered arm and eye trajectories, and demonstrate greater reliance on visual feedback (Borne et al., 2024; Paizis et al., 2008). In other words, previously automized motor tasks begin to heavily rely on cognitive resources and sensory feedback. This required shift in neural control strategies to maintain motor performance becomes more apparent in the context of non-standard interactions that decouple the actions of the eyes and hand while simultaneously requiring the integration of rules to perform the movement. When performing dissociated eye-hand visuomotor transformation tasks, older adults are slower than their younger counterparts and demonstrate longer and more complex eye scanning patterns (Baugh and Marotta, 2009). Several studies have further demonstrated age-related impairments in adaptive shifts across different decoupled visuomotor rotation tasks, but not in movement after-effects (Bock, 2005; Bock and Girgenrath, 2006; McNay and Willingham, 1998), suggesting that it is the strategic or rule-based aspect of these forms of movements that is especially sensitive to age-related degradation, as opposed to the basic sensorimotor transformation. Normal age-related declines in cognition and motor control have been partly attributed to changes in the structural and functional substrates of these abilities, notably in the prefrontal cortex, premotor regions, and cerebellum. In particular, structural brain imaging studies indicate a loss of cortical grey and white matter among aging adults (Raz, 1997; Salat, 2004), while functional imaging studies demonstrate increased frontal activity that has been related to inefficient and/or noisy processing (Turner and Spreng, 2012). There is increasing evidence that subtle functional declines may begin as early as midlife, and that this time period may be prognostic of brain health in old age (Kivipelto et al., 2006; Zhou et al., 2023). Yet, this age group remains relatively understudied, particularly in the context of multidomain integration.

As the neurological system undergoes a transition from one state of developmental aging to another in midlife, neuroendocrine changes occur in the background. Although it is typically considered from a reproductive perspective, hormonal status inherently alters neural processes and functioning, and consequently, behaviour. In adult females of reproductive age, fluctuations in estradiol and progesterone have been shown to affect processing speed, attention, and verbal and visual memory (Hampson and Kimura, 1988; Hatta and Nagaya, 2009). Interestingly, these differences do not always persist when comparing by menstrual cycle phase (Jang et al., 2025). Conversely, some studies have shown an influence of both the menstrual cycle and endogenous estrogen on sensory perception, fine motor skills, and executive functioning (Bayer and Hausmann, 2012; Colzato et al., 2012; Ho et al., 1986; Ocklenburg et al., 2013). In addition, higher activity in frontal regions during verbal memory tasks has been related to increased levels of estradiol (Joffe et al., 2006; Shaywitz, 1999). During the luteal phase, when levels of progesterone are elevated, increased resting-state connectivity has been reported, along with higher activity in the right dorsolateral prefrontal cortex and bilateral striatum during spatial tasks (Hidalgo-Lopez and Pletzer, 2021; Konrad et al., 2008; Pletzer and Harris, 2018). As females transition from peri- into post-menopause, steroid hormones sharply decline, resulting in persistent disruptions to these abilities (Kaur, 2025; Lissaman et al., 2024). In males and females, testosterone has been related to mental rotation and spatial ability (Cherrier et al., 2001, 2007; Young et al., 2010), as well as to motor performance (Killanin et al., 2023). Like estradiol and progesterone, testosterone levels decline in midlife, and this has been associated with cognitive changes (Beauchet, 2006) and motor dysfunction (Fargo et al., 2007). Collectively, these findings suggest that levels of sex steroid hormones alter cognitive and sensorimotor functioning, and this aligns with the timeframe in which subtle functional declines may begin. What is missing to date, however, is a characterization of standard and non-standard eye-hand movement as working-aged adults progress into middle- and retirement age, and whether this process is related to fluctuations in steroid hormones. In addition, earlier work has generally indirectly investigated hormone status (i.e., by looking at menstrual cycle phase or menopausal status) and often includes only one sex. Therefore, the purpose of the present study was to expand on this work by examining how levels of steroid hormone affect performance on a rule-based visuomotor task in working-aged males and females, and whether these hormones affect the structure of brain networks underlying this performance. We hypothesized that both age and hormone levels would impact performance on both simple eye-hand coordination and on rule-based tasks, and identify which factor (hormonal status or chronological age) would more strongly account for variability in behaviour. A secondary, exploratory aspect of the study was to examine whether hormone-related performance differences are related to structural changes in brain regions responsible for complex motor control.

## 2 Methods

### 2.1 Participants

Thirty-six working-aged adults with no cognitive impairments were included in this study (18 female, 18 male; see Table 1 for demographic information and Table 2 for hormone measures). Participants were asked to self-report sex on a binary scale (female/male). All participants were recruited from the community. Testing took place at York University between 2022 and 2024. Exclusion criteria included uncorrected visual impairments, upper-limb impairments, any medical conditions that would hinder motor task performance (e.g., severe arthritis or dystonia), neurological illnesses (e.g., Parkinson’s disease, depression, schizophrenia, alcoholism, epilepsy), current head injury (e.g., mild, severe), stroke, and any kind of birth control or hormonal therapy. The study protocol was approved by the Human Participants Review Sub-Committee of York University’s Ethics Review Board.

**Table 1:** Participant demographic features.

Feature	Males	Females	Combined males and females
*n*	18	18	36
Age (range)			45.50 (30–65)
Highest level of Education			
High school			2
College			3
University			15
Postgraduate			14
Unreported			2

**Table 2:** Participant hormone concentrations.

Hormone concentration	Males	Females	Combined male and female
Mean estradiol concentration (pg/ml) ± SD	1.21 ± 0.65	1.67 ± 0.79	1.44 ± 0.76
Mean progesterone concentration (pg/ml) ± SD	90.08 ± 53.08	109.38 ± 84.86	99.73 ± 70.44
Mean testosterone concentration (pg/ml) ± SD	173.76 ± 77.41	91.37 ± 68.34	133.07 ± 82.96

### 2.2 Saliva collections

To determine levels of 17β-estradiol, progesterone, and testosterone, a total of 1.5 mL of saliva was collected from each participant in microtubes and stored in a freezer at −80 °C. Salivary concentrations of steroid hormones were measured using the Salimetrics enzyme-linked immunosorbent assay (ELISA) kits (Kit Lot numbers 1–3,702, 1–1,502, and 1–2,402, respectively).

### 2.3 Behavioural data

All participants performed two visuomotor transformation tasks. The tasks involved simple sliding finger movements between targets displayed on an ASUS touchscreen tablet mounted in a vertical orientation (Asus Transformer Book T100TAF) or a Dell touchscreen laptop (Dell Latitude 5,511), both of which were externally connected to a USB touchpad (Keytec, TycoTouch, USA) situated perpendicular (in the horizontal plane) to the vertical screen. In both visuomotor tasks, participants were instructed to slide the finger of their dominant hand to displace a cursor from a central target towards one of four peripheral targets (top, bottom, left, right) as quickly and accurately as possible.

**Figure 1:**
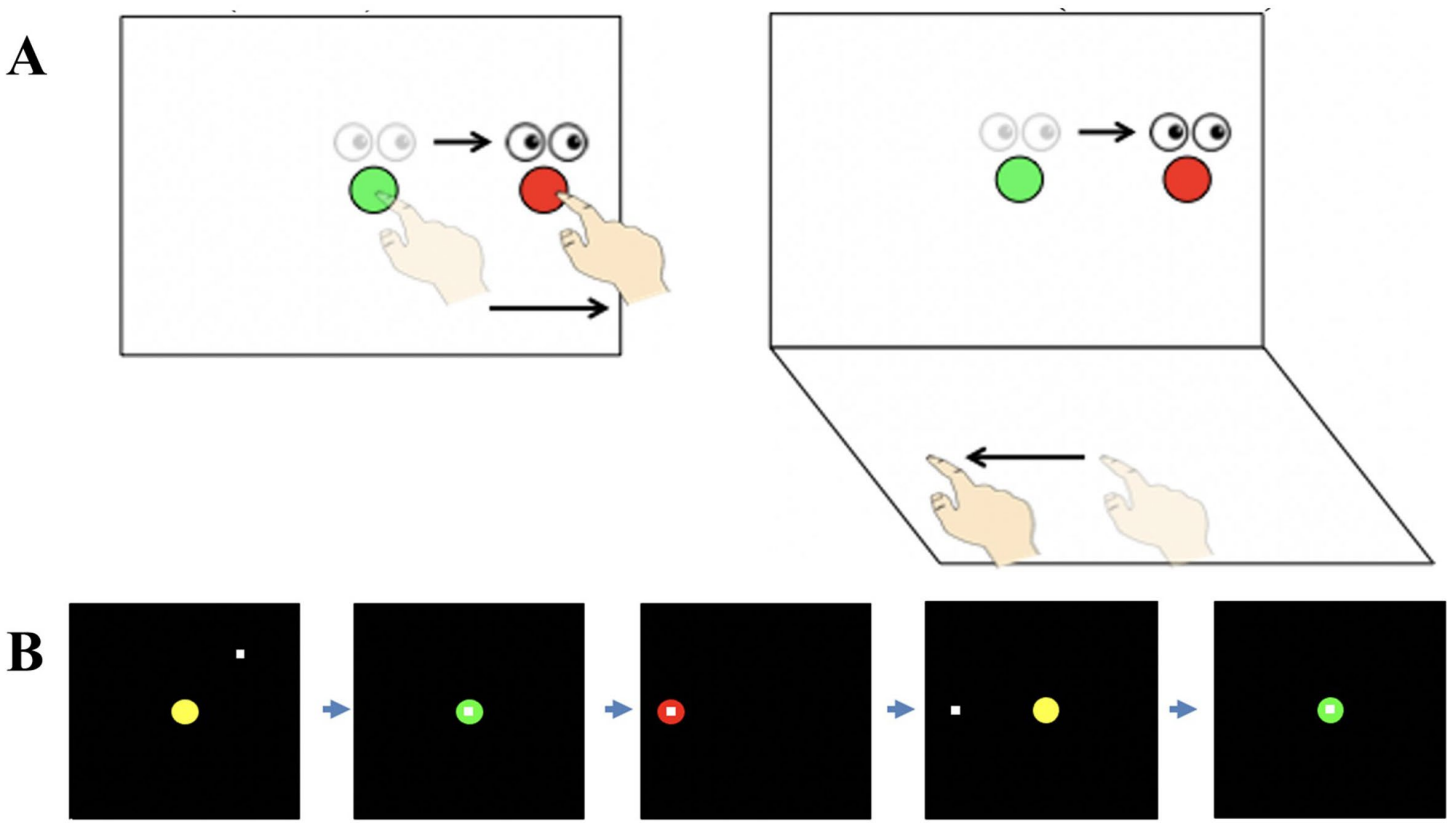
**(A)** Schematic drawing of the visuomotor task conditions. Lighter eye and hand symbols denote the starting position for each trial (green central target). Darker eye and hand symbols denote the instructed eye and hand movements for each task. Red circles represent the peripheral (reach) target, presented randomly in one of four locations (left, up, right, or down relative to the central target). The direct interaction task (left panel) requires standard mapping, where participants slide their finger on a touch screen to move a cursor from a central target to one of four peripheral targets. The non-standard task (right panel) requires cognitive-motor integration and involves indirect interactions with the target. Targets had a 180° feedback reversal and were spatially dissociated from the plane of hand motion (plane-change). **(B)** Sequence of events during one trial of the visuomotor task. Once the central (yellow) target appears, participants move the cursor (denoted by the white square) into the central target. Next, the target changes from yellow to green to signify a movement preparation period. After 2,000 ms, a red peripheral target appears in one of four directions (up, down, left, or right of the centre) and serves as the ‘go’ signal. Once the peripheral target is reached and is held for 500 ms, it disappears, signaling the end of the trial. After an inter-trial interval of 2,000 ms, the central yellow target reappears, and the participant moves back to the central target to initiate the next trial. Figure adapted from Rogojin et al. (2022).

Conditions were presented in randomized blocks, each consisting of five trials to each of the four targets, presented in random order, for a total of 20 trials per condition. The peripheral targets were located 75 mm from the central target, with target diameters set to 20 mm. The tasks were displayed on a 170 × 170 mm black square and a surrounding grey background. At the beginning of each trial, a central yellow target appeared, cueing participants to slide a white cursor to its centre, changing its colour to green once they arrived. After holding the central target for 4,000 ms, one of four peripheral red targets appeared and the central target disappeared, serving as the ‘go’ signal for participants to initiate their movement. Participants were told to look towards the visual target and slide their finger along the touchscreen to direct the cursor towards the target; once the peripheral target was reached and held for 500 ms, it disappeared, signaling the end of the trial. The next trial began with the presentation of the central target after an inter-trial interval of 2,000 ms (see Figure 1 for visual representations of a single trial completion).

Two conditions were performed. In the standard (direct) condition, the spatial location of the viewed target and required movement were the same (i.e., participants were looking at and moving their hand on the vertical screen). The non-standard condition was a cognitive-motor integration (CMI) task that involved two levels of decoupling. Targets were spatially dissociated from the plane of hand motion, meaning participants viewed targets in the vertical plane, but had to slide their finger on the horizontal touchpad placed directly below the vertical screen. Visual feedback for this condition was also rotated 180° (i.e., finger slides right, cursor moves left).

### 2.4 Behavioural data processing

Kinematic measures, including timing, finger position and error data, were recorded for each trial and converted into a MATLAB binary readable format using a custom-written (C++) application. Custom analysis software (MATLAB, Mathworks Inc.) was used to process finger trajectories and generate a computerized velocity profile of each trial’s movement. Movement onset and ballistic movement offsets were scored at 10% peak velocity, while total movement offsets were scored as the final 10% peak velocity point once the finger was in the correct peripheral target. If the initial movement successfully resulted in the finger reaching the peripheral target, ballistic and total movement offsets were equivalent. These movement profiles were then verified by visual inspection and were manually corrected if necessary. Unsuccessful (error) trials were detected by the data collection software and resulted in trial termination if the finger left the home target too early (<2,000 ms), reaction time (RT) was too short (< 150 ms), RT was too long (> 8,000 ms), or total movement time was too long (> 10,000 ms). Trials in which the first ballistic movement exited the boundaries of the central target in the wrong direction (greater than 45° from a straight line to the target) were coded as direction reversal errors, and analyzed as separate variables from the correct trials. All scored data were then processed to compute 7 different kinematic timing and path measures. Any trials exceeding 2 standard deviations from the participant’s mean for any of the outcome measures were eliminated from final outcome calculations. Performance across all correct trials was then averaged for each outcome measure, in both conditions.

The movement timing variables were as follows: (1) Reaction time (RT), the time interval (in ms) between the central target disappearance and movement onset; (2) Full movement time (MT), the time (in ms) between movement onset and offset, and (3) Peak velocity (PV), the maximum velocity (in mm/ms) obtained during the ballistic movement, and used to calculate the 10% threshold used for determining movement onsets and offsets for each trial. The path variables were: (1) Full pathlength (PL), the total distance (in mm) between the start and final end location of the cursor movement; (2) Absolute error (AE), or endpoint accuracy, measured as the distance (in mm) between the mean individual movement end points and the actual target location (defined as its center); (3) Variable error (VE), or end point precision, calculated as the distance (in mm) between the end points of individual movements (σ2) from their mean movements; and (4) Percentage direction reversals (%DR) recorded as a percentage of total completed trials resulting in this error. To directly compare differences between the standard and non-standard CMI conditions, performance in the standard condition was subtracted from performance in the non-standard condition to create ‘change’ scores (i.e., ΔRT, ΔMT, etc.).

### 2.5 Magnetic resonance imaging

#### 2.5.1 Image acquisition

MRI data were acquired using a 3 Tesla (3 T) Siemens PrismaFit scanner at York University. Participants received a T1-weighted anatomical scan using a sagittal volumetric magnetization-prepared rapid gradient echo (MP-RAGE) sequence. The MP-RAGE consisted of the following acquisition parameters: 192 sagittal slices (slice thickness of 1 mm, with no gap), field of view (FOV) of 256 × 256 mm, matrix size of 256 × 256 resulting in a voxel resolution of 1 × 1 × 1 mm, echo time (TE) = 2.96 ms, repetition time (TR) = 2,300 ms, flip angle = 9°. The MP-RAGE scan was used to quantify grey matter volume and thickness.

Twenty-six of the participants were included in a separate study examining the functional correlates of simple and complex visuomotor transformations. Therefore, these participants lay supine in the scanner with their heads tilted forward approximately 30° using a plastic wedge placed under the head coil to allow direct viewing and interactions with a target. Tilting participants’ heads for direct viewing of targets for movement required the use of the bottom half of a 32-channel receive-only head coil at the back of the head (integrated into the head cradle) with a 4-channel flex coil over the forehead to collect signal from the anterior part of the brain (Chen et al., 2022; Gorbet and Sergio, 2016). The remainder of the participants lay completely supine, without having their heads tilted. Importantly, during the T1 scans used to acquire cortical thickness measures, participants lay idle and did not perform a task.

#### 2.5.2 Structural MRI

Preprocessing: To compensate for reduced signal in the anterior part of the brain from participants in the flex coil setup, the T1-weighted images that were acquired with the head tilted were denoised using the BM4D denoising algorithm (Dabov et al., 2007; Maggioni et al., 2013). Tian et al. (2021) provided evidence that image denoising techniques have the potential to improve surface reconstruction in sub-millimetre resolution T1-weighted images; therefore, BM4D denoising was performed assuming Rician noise with an unknown noise standard deviation and was set to estimate the noise standard deviation and perform collaborative Wiener filtering with the “modified profile” option using the publicly available MATLAB-based software.^1^ The denoised images were then ACPC aligned to facilitate Talairach transformations in subsequent processing steps. These denoising and ACPC alignment steps were not applied to the T1-weighted scans that were acquired in the standard supine position with the full 32-channel head coil.

All anatomical scans were processed, and the cortical surface was reconstructed using FreeSurfer 7.1 (Harvard Medical School, Boston, USA; https://surfer.nmr.mgh.harvard.edu/) with individual T1-weighted MR images serving as input. The main Freesurfer reconstruction pipeline (“*recon-all*”) was used to parcellate and segment the brain into anatomically distinct regions of the cortex and subcortical nuclei, respectively. Visual inspection of each participant’s parcellation and segmentation was performed to ensure that there were no obvious errors, including excessive motion or signal dropout.

Regions of interest (ROIs) were chosen because of their involvement in visuomotor processing. This basic frontoparietal reach network consists of the parietooccipital region, superior parietal lobule (SPL), inferior parietal lobule (IPL), primary and secondary somatosensory cortices, and primary motor cortex. Dissociated movements additionally involve activity in the prefrontal cortex, medial premotor cortex, cuneus, middle occipital gyrus, fusiform gyrus, temporal cortex, and cerebellum. Thus, the selected ROIs included: bilateral frontal pole (FP), superior frontal gyrus (SFG), rostral/caudal middle frontal gyrus (MFG), inferior frontal gyrus (IFG) pars triangularis and opercularis, precentral gyrus (preCG), postcentral gyrus (postCG), superior parietal cortex, inferior parietal cortex, anterior/posterior supramarginal gyrus (SMG), precuneus, cuneus, superior temporal gyrus (STG), middle temporal gyrus, inferior temporal gyrus and temporooccipital part (ITG), temporal pole, fusiform cortex, superior/inferior lateral occipital cortex (LOC), rostral/caudal anterior and posterior cingulate cortex, and the right/left cerebellar cortices.

Grey matter volume and thickness: Volume and thickness measures for our ROIs were obtained from the Desikan-Killiany atlas (Desikan et al., 2006) used in *recon-all*.

Intracranial volume adjustment: Intracranial volume (ICV) adjustment is an important step as it takes into account individuals’ variations in head size. ICV was calculated from the T1-weighted images using Statistical Parametric Mapping (SPM) 12 software. Our cortical regions of interest were ICV-corrected using the residuals method. This method was originally described by Mathalon et al. (1993) and aims to remove the ICV-VOI relationship. Cortical thickness was not normalized as thickness measures do not scale linearly with head size.

### 2.6 Statistical analyses

The primary aim of this study was to investigate the relationship between steroid hormone concentrations and rule-based visuomotor performance. Sex differences in cognition and motor control are well known and established. With respect to tasks that require the integration of cognitive rules into movement planning, earlier work has documented sex differences, but this has generally been in the context of neurodegeneration or trauma (Rogojin et al., 2019; Sergio et al., 2020). Importantly, we have previously shown that even when performance is equivalent between males and females, sex differences exist in the neural networks controlling this performance (Gorbet et al., 2010; Gorbet and Sergio, 2007). Thus, while the task employed in this study has been shown to be sensitive to sex in terms of neural control networks, we were interested in the potential mechanisms underlying these differences. It has been previously suggested that steroid hormone levels may be the basis for sex differences in physiology and performance (Handelsman et al., 2018), and that exposure to hormone levels across the lifespan may organize neural circuits for male-typical and female-typical behaviours (Schulz and Sisk, 2006; Tyborowska et al., 2024; Wu et al., 2009). There were therefore two goals to our analysis: (1) examine whether performance is affected by sex, and (2) determine whether sex hormone concentrations may predict such performance differences. We first performed an independent-samples t-test to compare whether visuomotor performance differed between males and females. Next, hierarchical multiple linear regressions were performed to determine if age and steroid hormone concentrations could significantly predict the performance of the kinematic dependent variables in both the standard and CMI conditions. Because sex and sex hormone concentrations are highly correlated, our sample size is relatively small, and our primary factor of interest was steroid hormones and their impact on movement control, sex was not included in this analysis. To examine the different factors that may contribute to visuomotor performance in healthy working-aged adults, linear regressions were performed, beginning with age in the first block, followed by one of the three steroid hormones in the second block (estradiol, progesterone, and testosterone). Last, we aimed to explore whether these relationships depend on structural measures in regions known to be important for visuomotor ability. Regression models were set up with steroid hormones as the predictor variable, visuomotor performance measures as the dependent variable, grey matter measures as the moderating variable, and were controlled for age. The *p*-values were adjusted for multiple comparisons using false discovery rate (FDR) adjustments and were considered statistically significant at *p* < 0.05. For example, a regression model examining whether testosterone is predictive of movement time in the non-standard condition, moderated by frontal pole (FP) thickness:


$$\text{Movement time}=\beta_{1}\text{Testosterone}+\beta_{2}\mathrm{FP}+\beta_{3}\text{Testosterone}\cdot \mathrm{FP}+\beta_{0}$$

If a significant interaction was identified, simple slopes analyses were used to examine the effects of hormone concentrations on performance within ROI thickness values of ± 1 SD.

Outliers were identified as data values with z-scores greater than 3. These specific values were replaced with a value 2 standard deviations from the mean (Field, 2009). Three participants were excluded from the analyses examining performance in the standard condition and 1 participant was excluded from analyses examining non-standard performance due to missing behavioural data. Finally, two participants did not complete the MRI scan, and were consequently excluded from analyses assessing grey matter.

#### 2.6.1 Validity of cortical surface reconstruction using denoised images

Validity of the BM4D denoising prior to T1-weighted image cortical parcellation was assessed in a separate control study by comparing the volumes and thickness generated by the Desikan-Killiany atlas FreeSurfer recon-all cortical parcellation for each of our two MRI setups. Seven participants received two T1-weighted anatomical scans: one scan with their head in the tilted position using the 4-channel flex coil over their forehead with the posterior half of the 32-channel head coil behind their heads, and a second scan in a standard supine position using the full 32-channel head coil. BM4D denoising was applied only to the head tilt T1-weighted images. Denoised head tilt images were input into the FreeSurfer recon-all pipeline. T1-weighted images acquired using the full 32-channel head coil in a standard supine position were not denoised prior to input into the recon-all pipeline. Volume and thickness measurements were compared using intraclass correlation coefficients (ICC) based on a two-way random effects model with absolute agreement, as we were interested in consistency of output regional volumes and thicknesses between the two methods. Regions with an ICC of moderate strength and higher were included in the analysis (Desikan et al., 2006).

Statistical analyses were performed using SPSS statistical software (IBM Inc.) and open-source R software v4.1.0 (RCore Team, 2021).

## 3 Results

### 3.1 MRI preprocessing validity

Supplementary Table 1 lists the ICC for 34 parcellation region labels in each hemisphere. The average ICC for the comparison of the volumes using each MRI setup was 0.96, with values ranging from 0.63 (moderate) to 0.99 (excellent). The average ICC for the comparison of thickness measurements was 0.78, with values ranging from <0.001 (poor; left superior parietal cortex, excluded from the remaining analyses) to 0.99 (excellent). Of note, all ICCs for volume measurements were strong to excellent, except for the right caudal anterior cingulate cortex. ICCs for our ROI thickness measurements were generally strong to excellent. These findings suggest that the BM4D denoised T1 images can be used to obtain reliable cortical volume and thickness measurements.

### 3.2 Univariate analyses

**Figure 2:**
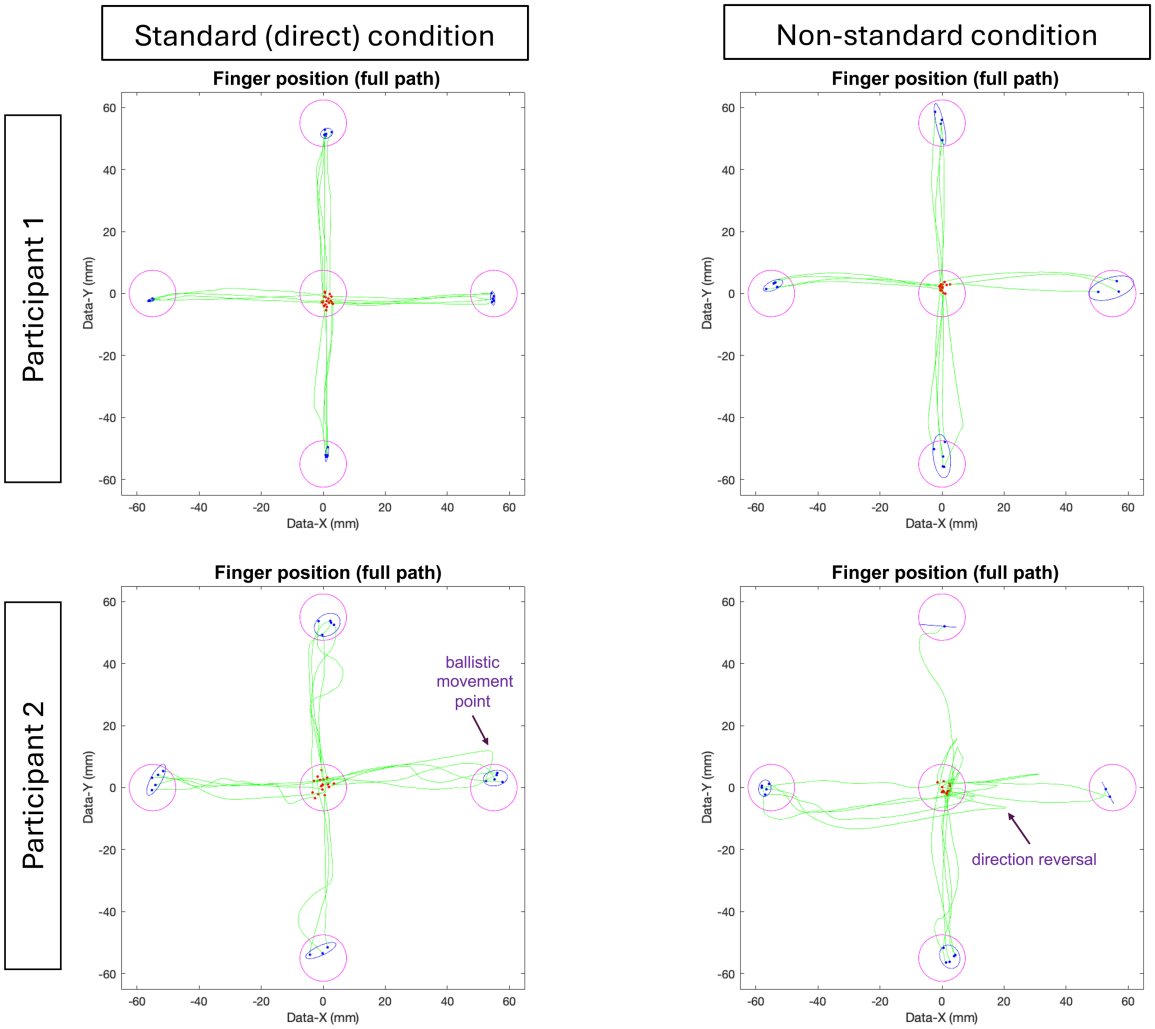
Examples of typical full hand movement trajectories for two participants in the standard and non-standard conditions. Trajectories begin at the central target (red dots) and move towards one of four peripheral targets, where each green line represents a single movement trajectory. Blue ellipses denote the 95% CI for the final endpoint of the finger movements (blue dots). Correct trials (green lines) are shown. Any target with fewer than 5 trajectories indicates error trials (not shown).

**Figure 3:**
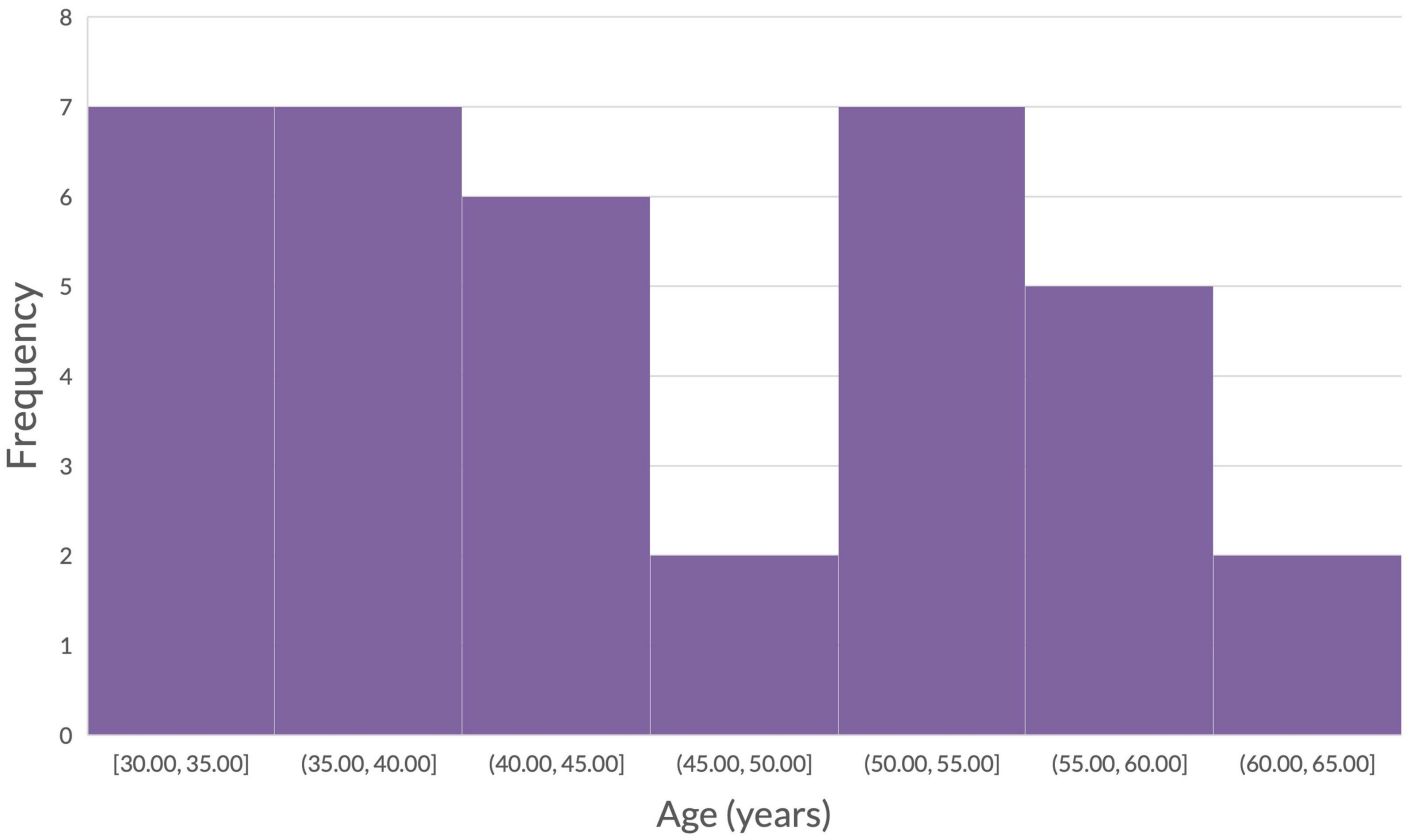
Distribution of participant ages.

Figure 2 presents a set of typical movement trajectories for both the standard and non-standard condition. Our participant sample consisted of healthy working-aged adults with a mean age of 45.5 years old (age distribution of the participants is depicted in Figure 3). Descriptive statistics of visuomotor kinematic variables are presented for all participants in Table 3.

**Table 3:** Descriptive statistics of visuomotor performance across all participants.

Kinematic variable	Standard condition mean (SEM)	Non-standard condition mean (SEM)	Δ mean (SEM)
Reaction Time (ms)	447.03 (15.10)	575.72 (40.68)	115.22 (36.32)
Movement time (ms)	568.40 (39.51)	1571.80 (198.32)	1044.42 (174.05)
Path length (mm)	3.00 (0.12)	10.21 (1.45)	11.69 (2.78)
Absolute error (mm)	2.83 (0.15)	5.48 (0.51)	7.52 (1.45)
Variable error (mm)	53.82 (0.28)	65.27 (2.89)	2.88 (0.52)
Peak Velocity (mm/ms)	125.03 (6.57)	85.12 (6.97)	−37.92 (6.10)
% Direction reversals	–	16.00 (2.97)	–

Behaviourally, 90.8% of the trials in the standard condition were successful across all participants, compared to 63.8% in the non-standard condition. Males demonstrated significantly larger accuracy (t = −2.57, *p* = 0.02) and precision errors (t = −2.12, *p* = 0.04; Table 4) in the standard condition. In the non-standard condition, males had significantly faster MTs (t = 3.17, *p* = 0.003), shorter PLs (t = 2.89, *p* = 0.007), and smaller AEs (t = 3.10, *p* = 0.004; Table 5). Finally, males demonstrated smaller differences in MT, PL, and AE across conditions (t = 2.68, *p* = 0.012; t = 2.66, *p* = 0.012; t = 3.32, *p* = 0.002, respectively; Table 6).

**Table 4:** Descriptive statistics of participant performance in the standard condition and statistical outcomes of the independent two-samples *t*-tests.

Kinematic variable	Males mean (SEM)	Females mean (SEM)	t (31)	*p*
Reaction Time (ms)	436.75 (19.07)	473.28 (24.96)	1.15	0.26
Movement time (ms)	497.38 (48.51)	651.65 (62.22)	1.94	0.06
Path length (mm)	53.46	54.20	1.26	0.22
Absolute error (mm)	3.31 (0.20)	2.71 (0.12)	−2.57	**0.02**
Variable error (mm)	3.15 (0.26)	2.54 (0.14)	−2.12	**0.04**
Peak velocity (mm/ms)	132.52 (7.50)	114.85 (10.88)	−1.32	0.20

Bold values emphasize measures that reached α = 0.05 significance level.

**Table 5:** Descriptive statistics of participant performance in the non-standard condition and statistical outcomes of the independent two-samples t-tests.

Kinematic variable	Males mean (SEM)	Females mean (SEM)	t (34)	*p*
Reaction Time (ms)	557.44 (34.55)	592.19 (66.18)	0.47	0.65
Movement time (ms)	1050.78 (1050.78)	2051.35 (240.35)	3.17	**0.003**
Path length (mm)	57.41 (2.13)	71.00 (4.20)	2.89	**0.007**
Absolute error (mm)	6.02 (0.45)	14.12 (2.58)	3.10	**0.004**
Variable error (mm)	4.66 (0.56)	5.99 (0.82)	1.34	0.19
Peak velocity (mm/ms)	97.93 (9.02)	73.16 (10.56)	−1.78	**0.042**
% Direction reversals	12.40 (4.20)	18.70 (4.29)	1.04	0.30

Bold values emphasize measures that reached α = 0.05 significance level.

**Table 6:** Descriptive statistics of differences in participant performance between the standard and non-standard condition and statistical outcomes of the independent two-samples *t*-tests.

Kinematic variable	Males mean (SEM)	Females mean (SEM)	t (31)	*p*
Δ Reaction time (ms)	110.94 (19.68)	124.41 (75.40)	0.168	0.87
Δ Movement time (ms)	600.94 (185.17)	1522.47 (284.44)	2.68	**0.012**
Δ Path length (mm)	4.45 (2.39)	19.18 (4.87)	2.66	**0.012**
Δ Absolute error (mm)	2.85 (0.42)	11.99 (2.64)	3.32	**0.002**
Δ Variable error (mm)	1.61 (0.51)	3.61 (0.88)	1.93	0.06
Δ Peak velocity (mm/ms)	33.37 (8.05)	40.42 (9.39)	−0.566	0.575

Bold values emphasize measures that reached α = 0.05 significance level.

### 3.3 No relationship between sex steroid hormone levels, age, and standard visuomotor performance

Blocked hierarchical regressions revealed that neither age nor concentrations of estradiol, progesterone, or testosterone were significantly associated with movement timing or trajectory variables during standard movement performance (all *p* > 0.05; Supplementary Tables 2–4).

### 3.4 Age and testosterone significantly predict non-standard movement performance

**Figure 4:**
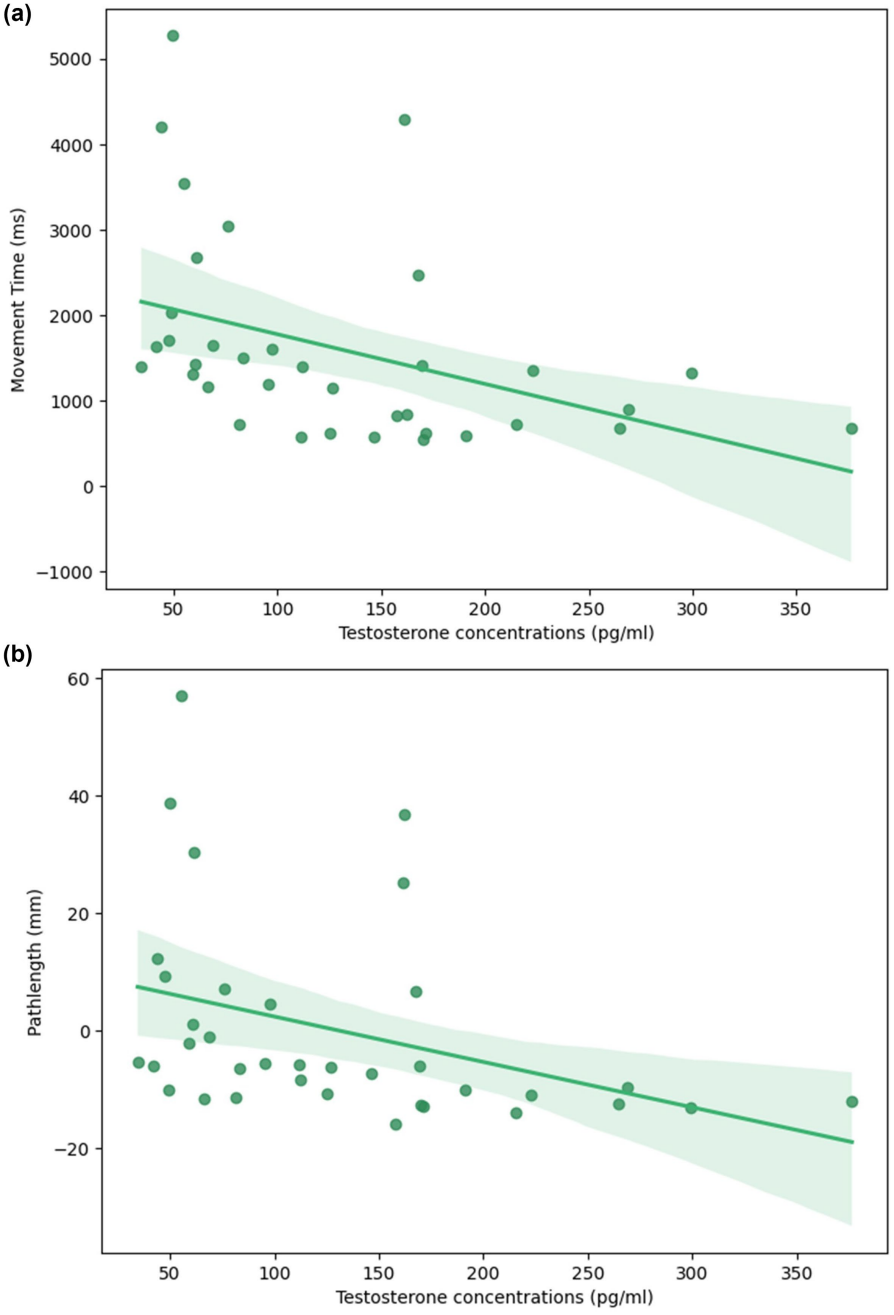
Relationship between testosterone concentrations and visuomotor performance in the non-standard condition. Higher levels of testosterone were associated with **(a)** faster movement times and **(b)** less variable pathlengths, after controlling for age. The solid line depicts the fitted linear regression and the shaded band its 95% confidence interval.

**Figure 5:**
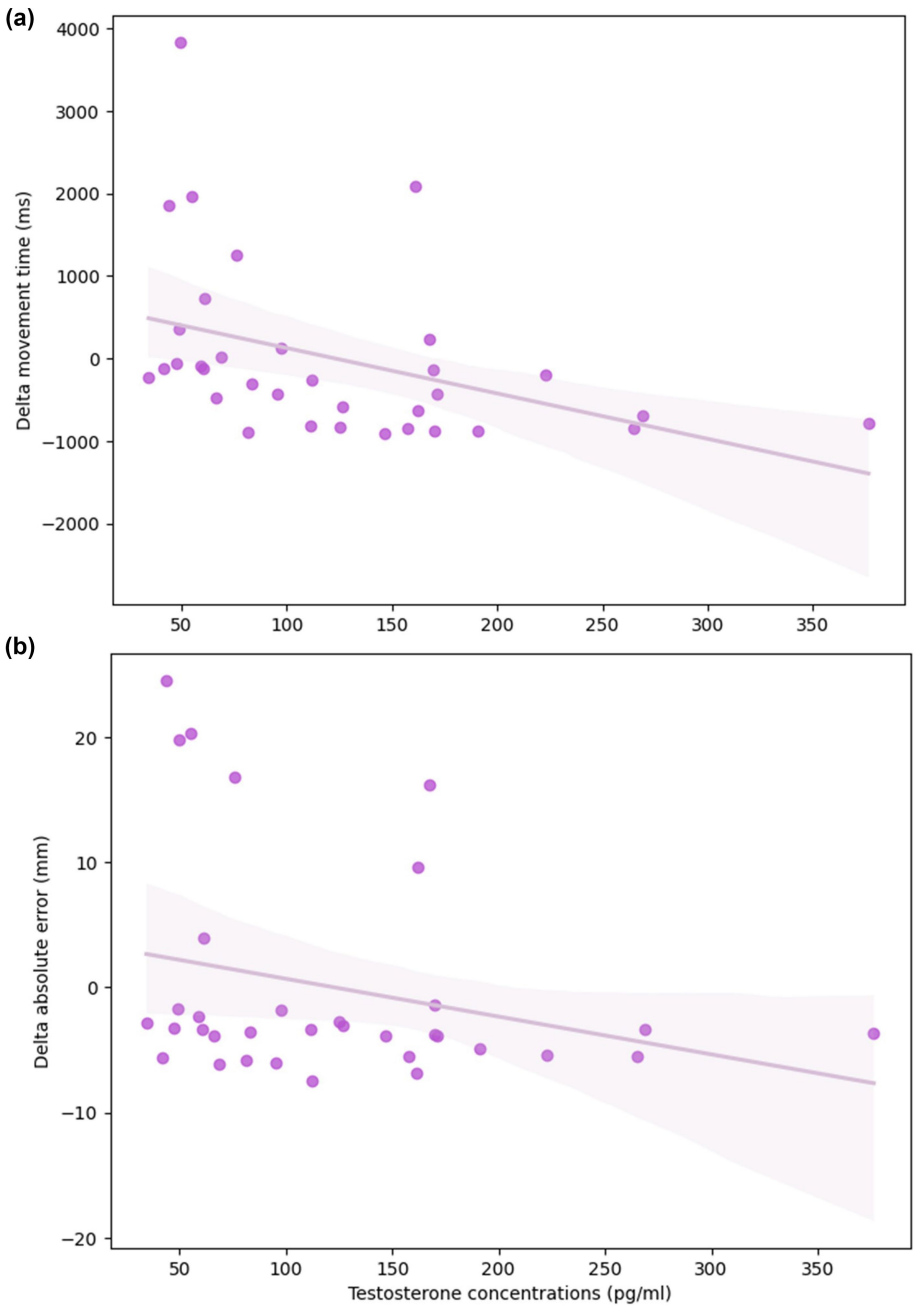
Difference in **(a)** movement time and **(b)** absolute error in the non-standard and standard conditions as a function of testosterone levels, after controlling for age; the shaded band represents the 95% confidence interval. Higher levels of testosterone are predictive of smaller differences in performance between conditions.

**Figure 6:**
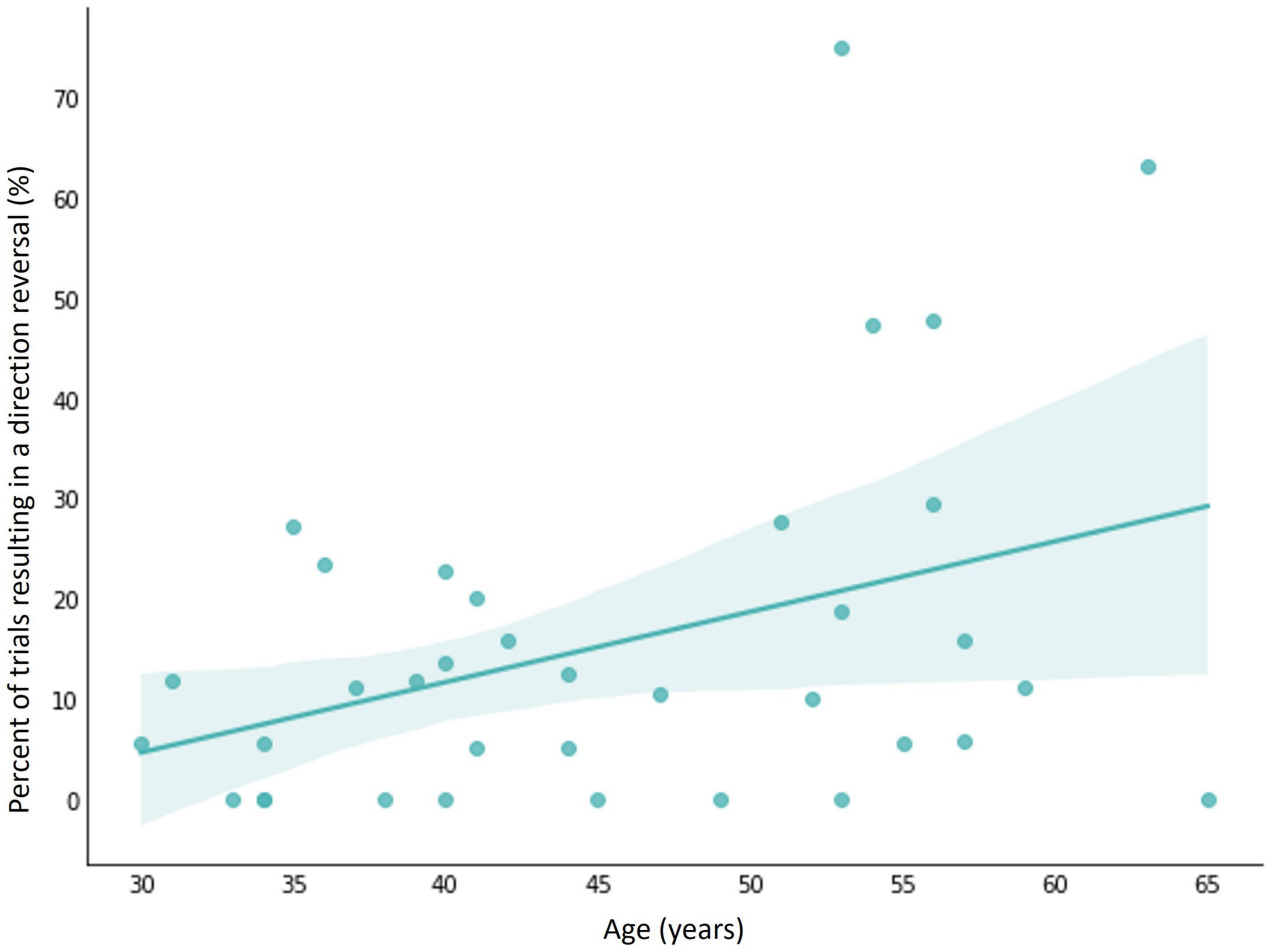
Relationship between age and the percentage of trials resulting in a direction reversal (%DR) in the non-standard CMI condition and shaded 95% confidence interval. Older age was predictive of a higher %DRs.

In the non-standard condition, we observed that levels of estradiol and progesterone were not associated with complex visuomotor performance or difference in performance between the standard and non-standard conditions (*p* > 0.05, Supplementary Tables 5–8). Notably, testosterone was the only steroid hormone significantly associated with complex movement. Specifically, MT in the non-standard condition was directly associated with concentrations of testosterone (Figure 4a; Table 7). Higher levels of testosterone were predictive of significantly faster MT, after controlling for age (ß = −5.50, *p* = 0.02, R^2^ = 0.147). Testosterone was also associated with PL in the non-standard condition (Figure 4b). After adjusting for age, individuals with greater concentrations of testosterone had shorter PLs (ß = −0.077, *p* = 0.029, R^2^ = 0.126). Testosterone concentrations were similarly associated with smaller ΔMT (ß = −5.52, *p* = 0.007, R^2^ = 0.169), ΔAE (ß = −0.037, *p* = 0.021, R^2^ = 0.124), and ΔVE (ß = −0.014, *p* = 0.015, R^2^ = 0.171; Table 8), indicating smaller differences in performance between conditions (Figures 5A,B). Finally, the %DRs in the non-standard condition was directly related to age. Individuals experienced a greater change in %DRs every year aged (ß = 0.785, *p* = 0.006, R^2^ = 0.189, Figure 6).

**Table 7:** Association between non-standard visuomotor performance, age, and concentrations of testosterone.

Kinematic variable	Model	Predictor	Unstandardized β	*p*-value	*R^2^*
Reaction Time (ms)	Model 1	Age	3.872	0.324	0.026
	Model 2	Age	4.925	0.237	0.044
		Testosterone (pg/ml)	0.410	0.415	
Movement time (ms)	Model 1	Age	9.610	0.613	0.007
	Model 2	Age	−4.523	0.810	0.147
		Testosterone (pg/ml)	−5.502	**0.020**	
Path length (mm)	Model 1	Age	−0.062	0.827	0.001
	Model 2	Age	−0.260	0.360	0.126
		Testosterone (pg/ml)	−0.077	**0.029**	
Absolute Error (mm)	Model 1	Age	0.033	0.814	0.002
	Model 2	Age	−0.050	0.730	0.089
		Testosterone (pg/ml)	−0.032	0.071	
Variable Error (mm)	Model 1	Age	−0.063	0.212	0.042
	Model 2	Age	−0.089	0.091	0.107
		Testosterone (pg/ml)	−0.010	0.114	
Peak Velocity (mm/ms)	Model 1	Age	0.058	0.937	0.000
	Model 2	Age	0.100	0.901	0.001
		Testosterone (pg/ml)	0.012	0.900	
% Direction reversals	Model 1	Age	0.804	**0.004**	0.201
	Model 2	Age	0.754	**0.011**	0.208
		Testosterone (pg/ml)	−0.020	0.568	

Bold values emphasize measures that reached α = 0.05 significance level.

**Table 8:** Association between the difference in standard and non-standard visuomotor performance, age, and concentrations of testosterone.

Kinematic variable	Model	Predictor	Unstandardized β	*p*-value	*R^2^*
Δ Reaction time (ms)	Model 1	Age	1.554	0.688	0.004
	Model 2	Age	2.992	0.485	0.020
		Testosterone (pg/ml)	0.416	0.421	
Δ Movement time (ms)	Model 1	Age	8.355	0.582	0.007
	Model 2	Age	−9.685	0.531	0.169
		Testosterone (pg/ml)	−5.222	**0.007**	
Δ Path length (mm)	Model 1	Age	−0.228	0.476	0.012
	Model 2	Age	−0.473	0.173	0.079
		Testosterone (pg/ml)	−0.071	0.093	
Δ Absolute Error (mm)	Model 1	Age	0.029	0.812	0.001
	Model 2	Age	−0.098	0.447	0.124
		Testosterone (pg/ml)	−0.037	**0.021**	
Δ Variable Error (mm)	Model 1	Age	−0.059	0.188	0.041
	Model 2	Age	−0.107	**0.024**	0.171
		Testosterone (pg/ml)	−0.014	**0.015**	
Δ Peak Velocity (mm/ms)	Model 1	Age	1.059	0.814	0.054
	Model 2	Age	0.729	0.338	0.079
		Testosterone (pg/ml)	−0.095	0.298	

Bold values emphasize measures that reached α = 0.05 significance level.

In summary, the results from these regression analyses emphasize the importance of testosterone in explaining visuomotor ability. Unexpectedly, except for %DRs, age was not a significant predictor of variance in movement performance among working-age adults.

### 3.5 Relationship between testosterone, grey matter measures, and non-standard visuomotor performance

To explore whether the observed hormone-related performance differences were moderated by grey matter thickness and volume, we conducted exploratory multiple regression analyses. Since testosterone was the only steroid hormone related to performance among working-aged adults, estradiol and progesterone were not included in these models. Additionally, we limited our analyses to measures of visuomotor performance that were significantly related to testosterone. Therefore, each model examined the influence of testosterone, ROI cortical volume/thickness measures, their interaction, and age on either non-standard movement time (MT) or path length (PL).

#### 3.5.1 Movement time

Right superior temporal volume was significantly associated with longer MT (ß = 0.97, *p* < 0.01, R^2^ = 0.45).

#### 3.5.2 Path length

There was a significant main effect of cortical thickness on PL in the non-standard condition, where individuals with higher cortical thickness in the right MTG, posterior cingulate (PC), and SFG had significantly shorter PLs (ß = −66.17, *p* = 0.045, R^2^ = 0.28; ß = −90.58, *p* = 0.039, R^2^ = 0.31; ß = −94.12, *p* = 0.024, R^2^ = 0.32, respectively). In addition, volumes in the bilateral caudal MFG and right preCG were significantly related to PL (left MFG: ß = 0.01, *p* = 0.023, R^2^ = 0.34; right MFG: ß = 0.02, *p* = 0.005, R^2^ = 0.38; right preCG: ß = 0.005, *p* = 0.04, R^2^ = 0.28, respectively).

**Figure 7:**
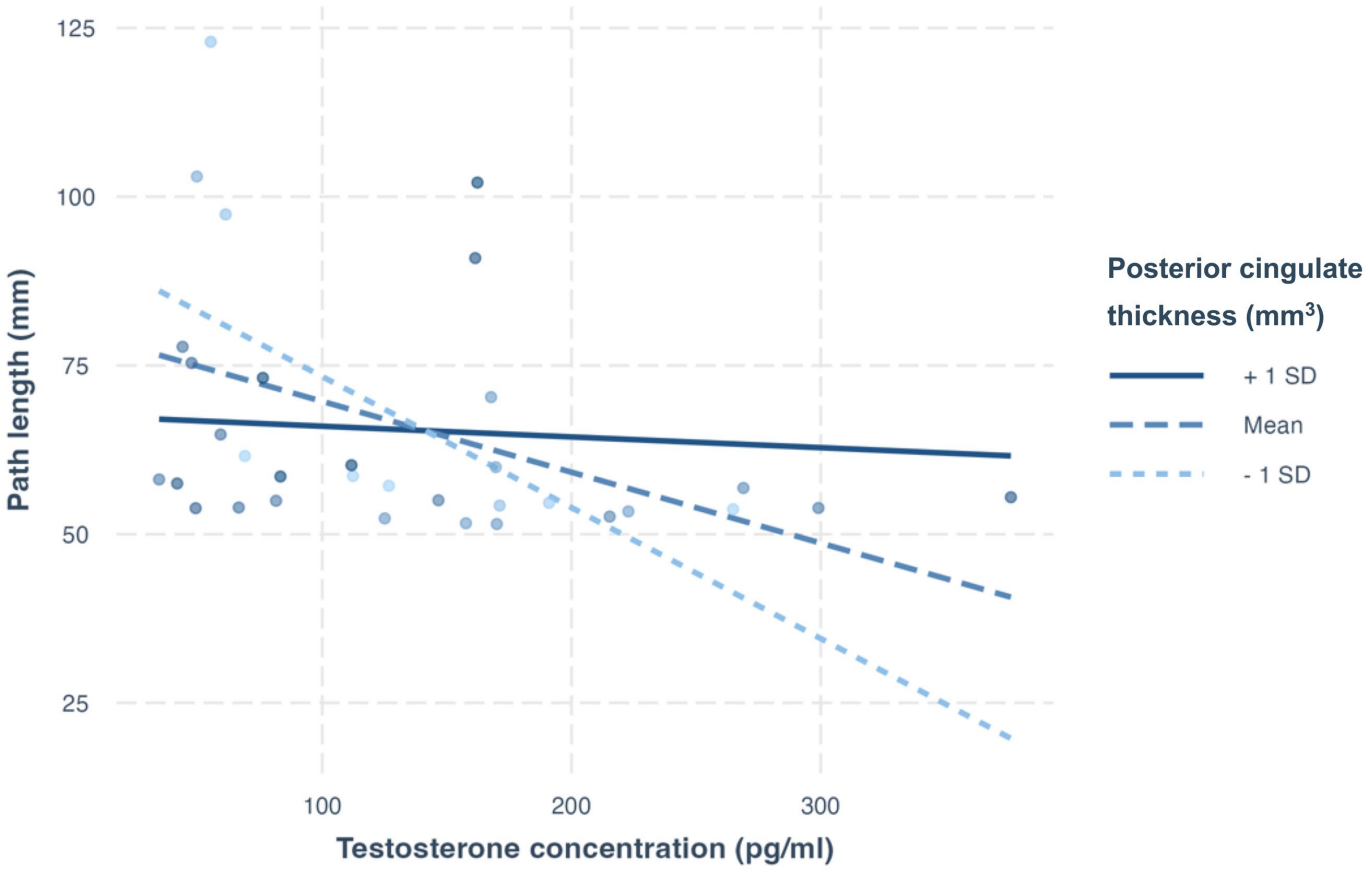
Simple slopes analysis from the testosterone-posterior cingulate cortex thickness interaction. Among individuals with mean to higher thickness in the right posterior cingulate, testosterone levels were associated with shorter path lengths, reflecting improved visuomotor performance. *p < 0.05.

There was a significant interaction of right PC thickness and testosterone on PL (ß = 0.64, *p* = 0.02, R^2^ = 0.31). Simple slopes analysis revealed that for individuals with higher right PC thickness (mean and +1 SD), testosterone was a significant predictor of shorter PLs (simple slope = −0.10, S.E. = 0.04, *p* = 0.01; simple slope = −0.19, S.E. = 0.06, *p* < 0.01, respectively; Figure 7).

Collectively, these results suggest that the relationship between testosterone levels and complex performance is related to grey matter thickness and volume in visuomotor control regions.

## 4 Discussion

This study sought to investigate the relationship between steroid hormones and visuomotor performance. A secondary goal was to assess whether hormone levels were related to neuroanatomy in regions important for visuomotor performance in males and females. Our key finding demonstrates that testosterone is the strongest predictor of rule-based, non-standard movement in healthy working-aged adults, and that this relationship is moderated by cortical thickness. Interestingly, estradiol and progesterone were not related to visuomotor performance, and a small effect of age was observed, contrary to our hypothesis that both of these factors would significantly affect visuomotor performance.

### 4.1 Testosterone improves non-standard visuomotor performance

Results from the present study verified sex differences in the performance of a cognitive-motor integration task, where males were faster and had shorter PLs. We’ve previously shown that sex is a significant predictor of performance in this task, among both healthy and clinical populations (Hawkins and Sergio, 2016; Rogojin et al., 2019; Smeha et al., 2022). Sex differences in motor skill performance have been documented more generally, including in studies examining eye-hand coordination (Dane and Erzurumluoglu, 2003; Gorbet and Staines, 2011; Chraif and Aniţei, 2013). In parallel, previous work has widely reported sex differences in cognitive domains important for CMI, including attention and spatial ability. Females are more affected by task-irrelevant information and perform more poorly during mental rotation tasks (Levine, 1997; Stoet, 2017). The present study aimed to expand on these findings by examining potential biological mechanisms that may underlie sex-based patterns of behaviours, such as hormone levels. Our salient results suggest that after accounting for age, testosterone is associated with enhanced performance in complex, rule-based movement. In particular, we observed that higher levels of the hormone predicted faster movement and less performance variability, as well as smaller changes in accuracy and precision, with increasing cognitive load. It is well known that concentrations of testosterone are higher in males compared to females (McEwen and Milner, 2017), and previous work has shown that females perform better on “male-dominated” tasks (i.e., those requiring spatial orientation and visualization) during periods of higher testosterone (Kimura, 2002; Hausmann et al., 2000; Pintzka et al., 2016). Testosterone levels have also been associated with improved working and visual memory in both males and females, as well as improved processing speed and visuospatial abilities in males (Janowsky, 2006; Moffat et al., 2002; Thilers et al., 2006). Adolescents with higher levels of testosterone similarly show improvements in fine motor skills (Killanin et al., 2023; Knatauskaitė et al., 2021; Wegner et al., 2014). In conjunction with our results, the body of evidence suggests that individuals with higher testosterone may have an enhanced ability to integrate cognitive rules into motor planning when performing decoupled movements.

The planning and execution of non-standard movements requires the activation of cortico-subcortical brain networks, particularly frontoparietal-cerebellar networks, in addition to intact connectivity between these regions via white matter tracts (Sergio et al., 2009; Kalaska et al., 1997; Battaglia-Mayer et al., 2001; Caminiti et al., 1999). The superior and inferior parietal lobules appear to be especially important for sensorimotor recalibration during decoupled visuomotor control, while the dorsolateral and ventral premotor and prefrontal regions are more relevant for strategic control (Granek et al., 2013; Granek and Sergio, 2015). Administration of a single dose of testosterone in healthy females has been shown to induce increased functional connectivity between the prefrontal and parietal cortices (Schutter et al., 2005), and greater activation of premotor regions has been observed in healthy males and females with higher serum testosterone during pain stimulation (Choi et al., 2006, 2011). Testosterone treatment has also been associated with thicker prefrontal and precuneal cortices (Burke et al., 2018). At the neural level, higher testosterone levels have been linked to greater arborization and dendritic spine density in the sensorimotor cortices (Chen et al., 2013; Stewart and Kolb, 1994), resulting in increased communication between neurons and coordination of information processing. The hormone has also been shown to modulate GABA receptor activity, support dopamine transporter activity, and is associated with larger motor neurons (Herbst and Bhasin, 2004; Killanin et al., 2023); these effects are linked to improved motor control. Finally, testosterone has been proposed to facilitate neuronal excitability and activity in the descending corticospinal pathways that control voluntary hand movements (Bonifazi et al., 2004). Because these regions in the visuomotor control network and peripheral nervous system abundantly express androgen receptors (Simerly et al., 1990; Abdelgadir et al., 1999; Goldstein et al., 2001), testosterone can both improve the structural integrity of the brain networks underlying complex movement and act as a neuromodulator. Therefore, individuals with higher levels of testosterone may have more efficient neural processing, a greater ability to recruit task-related regions, and facilitated neural transmission in descending pathways, ultimately resulting in improved performance. This is partially supported by our exploratory analysis, where testosterone levels were related to improved visuomotor performance among those with higher cortical thickness in the posterior cingulate. This region is involved in visual processing and has been implicated in non-standard visuomotor performance among cognitively healthy older adults with specific dementia factors (Rogojin et al., 2022). Thus, testosterone may directly or indirectly influence cortical thickness, and may thus result in skilled motor action. In contrast, standard movement, which represents the brain’s ‘default’ visuomotor mapping, requires less overall brain activation and may consequently be less reliant on the neuromodulatory properties of steroid hormones.

Several reasons may explain why we did not find the hypothesized relationships between visuomotor performance and estradiol or progesterone. The literature is abundant with conflicting findings with respect to the relationships between brain-based behaviours, menstrual cycle phase, and menopause (Conde et al., 2021; Jang et al., 2025; Sundström Poromaa and Gingnell, 2014), likely due to the drastic hormonal fluctuations that occur within and across each stage. More recent studies have begun to include concentrations of hormones, and some of this work has uncovered influences of estradiol and progesterone on cognition and movement (Dumas et al., 2010; Stein, 2005). Contrary to many of these studies, we examined a broader age and hormonal range than is typical (i.e., adults from working- into retirement-age, and females in their pre-, peri-, and post-menopausal stages), and included both sexes. Another important consideration is the fact that each domain is often independently studied, and hormone-related performance is generally investigated in the context of neurotrauma and neurodegeneration. With this in mind, we demonstrate that in the healthy state, the execution of rule-based, visually-guided movement may not directly involve progesterone and estradiol, but is instead facilitated by testosterone. Testosterone is the most abundant steroid hormone in both males and females, and subsequently, may have a larger neuromodulatory role in the processes underlying multidomain performance (Handelsman et al., 2018). This is substantiated by preliminary evidence from our group, which has shown relationships between non-standard task performance and testosterone levels in varsity female athletes, but no effect of ovarian hormones (Pierias, 2021), as well as unique effects of testosterone on the connectivity between brain regions supporting this ability. Although testosterone fluctuates and increases significantly during puberty, after this stage, levels of the hormone remain relatively stable across the lifespan. This is especially true when comparing variations in testosterone to those of estradiol and progesterone at different hormonal transition states in females (Dimitrakakis et al., 2002; Panay and Fenton, 2009; Scott and Newson, 2020). In addition, while all three hormones decline with age, levels of testosterone decline more gradually and are thus more stable. It is therefore perhaps only under conditions of biological stress, when instability triggers a shift from healthy states to dysfunctional ones, that estradiol and progesterone may be implicated.

### 4.2 Non-standard visuomotor performance and healthy aging

A small effect of age on non-standard visuomotor performance was observed in this study. In particular, older working-aged participants performed a greater number of direction reversals, which is reflective of deficits in movement planning. Several aspects of movement, including accuracy, speed, and planning ability, have been shown to worsen with age (Yan et al., 1998; Lamb et al., 2016). Age-related performance deficits in non-standard forms of movement are likewise well known (Baugh and Marotta, 2009; Hegele and Heuer, 2010). Many of these changes have been attributed to structural and functional changes in the frontal cortex. Indeed, normal aging appears to show a frontal dominance in its effects. This includes a reduction in overall grey matter volume and cortical grey matter thickness in frontal regions, a loss of white matter tracts in the frontal lobe but preservation in the posterior regions, and decreases in glucose metabolism in the frontal lobe (O’Sullivan et al., 2001; Pfefferbaum et al., 2005; Raz, 1997). Due to the importance of frontal regions for integrating implicit and explicit rules into motor planning, our observations may reflect early-stage changes in this region. Future work will examine the neural correlates of non-standard movement within this demographic because if such a relationship exists, non-standard tasks may offer a non-invasive and simple assessment to detect individuals who are at risk of later behavioural or neural dysfunction (Hawkins and Sergio, 2016).

### 4.3 General conclusions

Although research in the last decade has begun to take sex and steroid hormones into account, there remains a need to better understand how an individual’s hormone profile may affect cognitive-sensorimotor aspects of behaviour necessary for everyday living, particularly when these domains must be integrated. The findings presented here suggest that among healthy working-aged adults, testosterone is the strongest predictor of movements requiring the integration of sensory, cognitive, and motor information. In addition, we observed that age-related performance deficits, although small, begin to emerge in the working-age years. While the generalizability of these results is limited by the relatively small sample size, small statistical power limiting multivariate analyses, and cross-sectional study design, they provide important insights into individual processes that can influence skilled performance. An important open question that remains is whether these changes are reflected in functional brain activity during visuomotor processing, and whether hormone concentrations can provide resilience for age- and disease-related impairments. We suggest that an important next step is to include neuroendocrine status when taking into account personal differences, in order to comprehensively understand simple and complex movement control during healthy and pathological aging.

## Data Availability

The raw data supporting the conclusions of this article will be made available by the authors upon request, without undue reservation.

## Footnotes

1
https://www.cs.tut.fi/~foi/GCF-BM3D
