# Supplementary material for: Beyond sex: the effects of testosterone on visuomotor performance in men and women
Source: Front Hum Neurosci. 2026 Jan 12;19:1718846. doi: 10.3389/fnhum.2025.1718846 (PMC12833049; doi:10.3389/fnhum.2025.1718846)
Supplement: Supplementary file 1 [file Table_1.DOCX]

Supplementary Tables

**Table 1.** Intraclass correlations comparing volume and thickness measurements obtained from two different MRI setups

| Region | Left hem volume | Right hem volume | Left hem thickness | Right hem thickness |
| --- | --- | --- | --- | --- |
| Banks superior temporal sulcus | 0.98 | 0.98 | 0.94 | 0.92 |
| Caudal anterior-cingulate cortex | 0.99 | 0.63 | 0.89 | 0.87 |
| Caudal middle frontal gyrus | 0.99 | 0.97 | 0.95 | 0.93 |
| Cuneus cortex | 0.94 | 0.9 | 0.48 | 0.61 |
| Entorhinal cortex | 0.84 | 0.87 | 0.74 | 0.18 |
| Fusiform gyrus | 0.97 | 0.98 | 0.92 | 0.68 |
| Inferior parietal cortex | 0.98 | 0.97 | 0.27 | 0.6 |
| Inferior temporal gyrus | 0.94 | 0.95 | 0.56 | 0.54 |
| Isthmus–cingulate cortex | 0.97 | 0.99 | 0.95 | 0.96 |
| Lateral occipital cortex | 0.93 | 0.99 | 0.39 | 0.76 |
| Lateral orbital frontal cortex | 0.97 | 0.99 | 0.92 | 0.89 |
| Lingual gyrus | 0.97 | 0.96 | 0.72 | 0.89 |
| Medial orbital frontal cortex | 0.98 | 0.98 | 0.91 | 0.93 |
| Middle temporal gyrus | 0.97 | 0.97 | 0.91 | 0.61 |
| Parahippocampal gyrus | 0.96 | 0.97 | 0.87 | 0.91 |
| Paracentral lobule | 0.82 | 0.97 | 0.93 | 0.88 |
| Pars opercularis | 0.99 | 0.99 | 0.98 | 0.99 |
| Pars orbitalis | 0.99 | 0.97 | 0.86 | 0.91 |
| Pars triangularis | 0.99 | 0.99 | 0.58 | 0.94 |
| Pericalcarine cortex | 0.94 | 0.95 | 0.56 | 0.85 |
| Postcentral gyrus | 0.95 | 0.98 | 0.09 | 0.91 |
| Posterior-cingulate cortex | 0.99 | 0.97 | 0.94 | 0.52 |
| Precentral gyrus | 0.97 | 0.99 | 0.83 | 0.99 |
| Precuneus cortex | 0.95 | 0.98 | 0.45 | 0.6 |
| Rostral anterior cingulate cortex | 0.99 | 0.98 | 0.93 | 0.82 |
| Rostral middle frontal gyrus | 0.98 | 0.98 | 0.94 | 0.75 |
| Superior frontal gyrus | 0.99 | 0.97 | 0.97 | 0.47 |
| Superior parietal cortex | 0.88 | 0.96 | <0.001 | 0.97 |
| Superior temporal gyrus | 0.99 | 0.99 | 0.91 | 0.82 |
| Supramarginal gyrus | 0.98 | 0.99 | 0.71 | 0.82 |
| Temporal pole | 0.92 | 0.97 | 0.78 | 0.91 |
| Transverse temporal cortex | 0.9 | 0.91 | 0.9 | 0.97 |

**Table 2.** Association between standard visuomotor performance, age, and concentrations of estradiol.

|  | | Predictor | Unstandardized *β* | p-value | *R^2^* |
| --- | --- | --- | --- | --- | --- |
| Reaction Time (ms) | Model 1 | Age | 0.156 | 0.928 | 0.000 |
|  | Model 2 | Age | 1.079 | 0.544 | 0.081 |
|  |  | Estradiol (pg/ml) | 40.736 | 0.098 |  |
| Movement time (ms) | Model 1 | Age | -1.574 | 0.694 | 0.005 |
|  | Model 2 | Age | -0.400 | 0.924 | 0.029 |
|  |  | Estradiol (pg/ml) | 51.800 | 0.370 |  |
| Path length (mm) | Model 1 | Age | 0.009 | 0.754 | 0.003 |
|  | Model 2 | Age | 0.019 | 0.531 | 0.037 |
|  |  | Estradiol (pg/ml) | 0.437 | 0.289 |  |
| Absolute Error (mm) | Model 1 | Age | 0.002 | 0.875 | 0.001 |
|  | Model 2 | Age | -0.002 | 0.860 | 0.034 |
|  |  | Estradiol (pg/ml) | -0.188 | 0.294 |  |
| Variable Error (mm) | Model 1 | Age | 0.006 | 0.701 | 0.004 |
|  | Model 2 | Age | 0.003 | 0.841 | 0.013 |
|  |  | Estradiol (pg/ml) | -0.113 | 0.601 |  |
| Peak Velocity (mm/ms) | Model 1 | Age | -1.093 | 0.211 | 0.038 |
|  | Model 2 | Age | -1.403 | 0.121 | 0.076 |
|  |  | Estradiol (pg/ml) | -16.681 | 0.203 |  |

**Table 3.** Association between standard visuomotor performance, age, and concentrations of progesterone.

|  | | Predictor | Unstandardized *β* | p-value | *R^2^* |
| --- | --- | --- | --- | --- | --- |
| Reaction Time (ms) | Model 1 | Age | 0.179 | 0.915 | 0.000 |
|  | Model 2 | Age | 0.123 | 0.945 | 0.001 |
|  |  | Progesterone (pg/ml) | -0.021 | 0.916 |  |
| Movement time (ms) | Model 1 | Age | -0.963 | 0.806 | 0.002 |
|  | Model 2 | Age | -1.586 | 0.702 | 0.009 |
|  |  | Progesterone (pg/ml) | -0.231 | 0.612 |  |
| Path length (mm) | Model 1 | Age | 0.011 | 0.689 | 0.005 |
|  | Model 2 | Age | 0.016 | 0.596 | 0.013 |
|  |  | Progesterone (pg/ml) | 0.002 | 0.606 |  |
| Absolute Error (mm) | Model 1 | Age | 0.001 | 0.932 | 0.000 |
|  | Model 2 | Age | 0.002 | 0.882 | 0.002 |
|  |  | Progesterone (pg/ml) | 0.000 | 0.819 |  |
| Variable Error (mm) | Model 1 | Age | 0.005 | 0.713 | 0.004 |
|  | Model 2 | Age | 0.007 | 0.647 | 0.008 |
|  |  | Estradiol (pg/ml) | 0.001 | 0.707 |  |
| Peak Velocity (mm/ms) | Model 1 | Age | -1.001 | 0.243 | 0.032 |
|  | Model 2 | Age | -1.291 | 0.151 | 0.060 |
|  |  | Progesterone (pg/ml) | -0.116 | 0.275 |  |

**Table 4.** Association between standard visuomotor performance, age, and concentrations of testosterone.

|  | | Predictor | Unstandardized *β* | p-value | *R^2^* |
| --- | --- | --- | --- | --- | --- |
| Reaction Time (ms) | Model 1 | Age | 0.179 | 0.915 | 0.000 |
|  | Model 2 | Age | -0.112 | 0.950 | 0.009 |
|  |  | Testosterone (pg/ml) | -0.117 | 0.586 |  |
| Movement time (ms) | Model 1 | Age | -0.963 | 0.806 | 0.002 |
|  | Model 2 | Age | -2.550 | 0.531 | 0.050 |
|  |  | Testosterone (pg/ml) | -0.640 | 0.198 |  |
| Path length (mm) | Model 1 | Age | 0.011 | 0.689 | 0.005 |
|  | Model 2 | Age | 0.009 | 0.755 | 0.006 |
|  |  | Testosterone (pg/ml) | -0.001 | 0.827 |  |
| Absolute Error (mm) | Model 1 | Age | 0.001 | 0.932 | 0.000 |
|  | Model 2 | Age | 0.004 | 0.753 | 0.018 |
|  |  | Testosterone (pg/ml) | 0.001 | 0.437 |  |
| Variable Error (mm) | Model 1 | Age | 0.005 | 0.713 | 0.004 |
|  | Model 2 | Age | 0.009 | 0.535 | 0.028 |
|  |  | Testosterone (pg/ml) | 0.002 | 0.366 |  |
| Peak Velocity (mm/ms) | Model 1 | Age | -1.001 | 0.243 | 0.032 |
|  | Model 2 | Age | -0.629 | 0.503 | 0.054 |
|  |  | Testosterone (pg/ml) | 0.108 | 0.343 |  |

**Table 5.** Association between non-standard visuomotor performance, age, and concentrations of estradiol.

|  | | Predictor | Unstandardized *β* | p-value | *R^2^* |
| --- | --- | --- | --- | --- | --- |
| Reaction Time (ms) | Model 1 | Age | 3.866 | 0.339 | 0.025 |
|  | Model 2 | Age | 4.149 | 0.325 | 0.028 |
|  |  | Estradiol (pg/ml) | 16.394 | 0.776 |  |
| Movement time (ms) | Model 1 | Age | 8.965 | 0.647 | 0.006 |
|  | Model 2 | Age | 11.692 | 0.566 | 0.015 |
|  |  | Estradiol (pg/ml) | 158.035 | 0.573 |  |
| Path length (mm) | Model 1 | Age | -0.096 | 0.741 | 0.003 |
|  | Model 2 | Age | -0.017 | 0.953 | 0.037 |
|  |  | Estradiol (pg/ml) | 4.536 | 0.271 |  |
| Absolute Error (mm) | Model 1 | Age | 0.014 | 0.922 | 0.000 |
|  | Model 2 | Age | 0.039 | 0.795 | 0.014 |
|  |  | Estradiol (pg/ml) | 1.436 | 0.488 |  |
| Variable Error (mm) | Model 1 | Age | -0.073 | 0.155 | 0.055 |
|  | Model 2 | Age | -0.060 | 0.256 | 0.086 |
|  |  | Estradiol (pg/ml) | 0.774 | 0.283 |  |
| Peak Velocity (mm/ms) | Model 1 | Age | 0.129 | 0.861 | 0.001 |
|  | Model 2 | Age | 0.125 | 0.872 | 0.001 |
|  |  | Estradiol (pg/ml) | -0.252 | 0.982 |  |
| % Direction reversals | Model 1 | Age | 0.785 | 0.006 | 0.189 |
|  | Model 2 | Age | 0.783 | 0.009 | 0.189 |
|  |  | Estradiol (pg/ml) | -0.120 | 0.976 |  |

|  | | Predictor | Unstandardized *β* | p-value | *R^2^* |
| --- | --- | --- | --- | --- | --- |
| Reaction Time (ms) | Model 1 | Age | 3.872 | 0.324 | 0.026 |
|  | Model 2 | Age | 3.678 | 0.373 | 0.027 |
|  |  | Progesterone (pg/ml) | -0.078 | 0.863 |  |
| Movement time (ms) | Model 1 | Age | 9.610 | 0.613 | 0.007 |
|  | Model 2 | Age | 4.594 | 0.816 | 0.030 |
|  |  | Progesterone (pg/ml) | -2.006 | 0.357 |  |
| Path length (mm) | Model 1 | Age | -0.062 | 0.827 | 0.001 |
|  | Model 2 | Age | -0.068 | 0.819 | 0.001 |
|  |  | Progesterone (pg/ml) | -0.003 | 0.937 |  |
| Absolute Error (mm) | Model 1 | Age | 0.033 | 0.814 | 0.002 |
|  | Model 2 | Age | 0.047 | 0.754 | 0.005 |
|  |  | Progesterone (pg/ml) | 0.005 | 0.742 |  |
| Variable Error (mm) | Model 1 | Age | -0.063 | 0.212 | 0.042 |
|  | Model 2 | Age | -0.053 | 0.315 | 0.055 |
|  |  | Progesterone (pg/ml) | 0.004 | 0.479 |  |
| Peak Velocity (mm/ms) | Model 1 | Age | 0.058 | 0.937 | 0.000 |
|  | Model 2 | Age | -0.359 | 0.626 | 0.083 |
|  |  | Progesterone (pg/ml) | -0.166 | 0.061 |  |
| % Direction reversals | Model 1 | Age | 0.804 | 0.004 | 0.201 |
|  | Model 2 | Age | 0.703 | 0.014 | 0.240 |
|  |  | Progesterone (pg/ml) | -0.040 | 0.183 |  |

**Table 6.** Association between non-standard visuomotor performance, age, and concentrations of progesterone.

**Table 7.** Association between differences in standard and non-standard visuomotor performance, age, and concentrations of estradiol.

|  | | Predictor | Unstandardized *β* | p-value | *R^2^* |
| --- | --- | --- | --- | --- | --- |
| Δ Reaction Time (ms) | Model 1 | Age | 1.496 | 0.706 | 0.004 |
|  | Model 2 | Age | 0.659 | 0.873 | 0.017 |
|  |  | Estradiol (pg/ml) | -44.939 | 0.457 |  |
| Δ Movement time (ms) | Model 1 | Age | 8.433 | 0.587 | 0.007 |
|  | Model 2 | Age | 10.921 | 0.502 | 0.015 |
|  |  | Estradiol (pg/ml) | 133.743 | 0.572 |  |
| Δ Path length (mm) | Model 1 | Age | -0.259 | 0.427 | 0.015 |
|  | Model 2 | Age | -0.254 | 0.459 | 0.016 |
|  |  | Estradiol (pg/ml) | 0.282 | 0.955 |  |
| Δ Absolute Error (mm) | Model 1 | Age | 0.016 | 0.902 | 0.000 |
|  | Model 2 | Age | 0.049 | 0.708 | 0.023 |
|  |  | Estradiol (pg/ml) | 1.797 | 0.347 |  |
| Δ Variable Error (mm) | Model 1 | Age | -0.066 | 0.145 | 0.051 |
|  | Model 2 | Age | -0.051 | 0.268 | 0.082 |
|  |  | Estradiol (pg/ml) | 0.775 | 0.252 |  |
| Δ Peak Velocity (mm/ms) | Model 1 | Age | 1.222 | 0.080 | 0.073 |
|  | Model 2 | Age | 1.528 | **0.033** | 0.131 |
|  |  | Estradiol (pg/ml) | 16.429 | 0.111 |  |

**Table 8**. Association between difference in standard and non-standard visuomotor performance, age, and concentrations of progesterone.

|  | | Predictor | Unstandardized *β* | p-value | *R^2^* |
| --- | --- | --- | --- | --- | --- |
| Δ Reaction Time (ms) | Model 1 | Age | 1.554 | 0.688 | 0.004 |
|  | Model 2 | Age | 1.341 | 0.744 | 0.005 |
|  |  | Progesterone (pg/ml) | -0.085 | 0.861 |  |
| Δ Movement time (ms) | Model 1 | Age | 8.355 | 0.582 | 0.007 |
|  | Model 2 | Age | 5.281 | 0.741 | 0.018 |
|  |  | Progesterone (pg/ml) | -1.226 | 0.517 |  |
| Δ Path length (mm) | Model 1 | Age | -0.228 | 0.476 | 0.012 |
|  | Model 2 | Age | -0.252 | 0.457 | 0.014 |
|  |  | Progesterone (pg/ml) | -0.010 | 0.811 |  |
| Δ Absolute Error (mm) | Model 1 | Age | 0.029 | 0.812 | 0.001 |
|  | Model 2 | Age | 0.050 | 0.702 | 0.008 |
|  |  | Progesterone (pg/ml) | 0.008 | 0.594 |  |
| Δ Variable Error (mm) | Model 1 | Age | -0.059 | 0.188 | 0.041 |
|  | Model 2 | Age | -0.048 | 0.304 | 0.055 |
|  |  | Progesterone (pg/ml) | 0.004 | 0.441 |  |
| Δ Peak Velocity (mm/ms) | Model 1 | Age | 1.059 | 0.129 | 0.054 |
|  | Model 2 | Age | 0.932 | 0.204 | 0.062 |
|  |  | Progesterone (pg/ml) | -0.050 | 0.558 |  |
